# Endocrine features of Prader-Willi syndrome: a narrative review focusing on genotype-phenotype correlation

**DOI:** 10.3389/fendo.2024.1382583

**Published:** 2024-04-26

**Authors:** Simona F. Madeo, Luca Zagaroli, Sara Vandelli, Valeria Calcaterra, Antonino Crinò, Luisa De Sanctis, Maria Felicia Faienza, Danilo Fintini, Laura Guazzarotti, Maria Rosaria Licenziati, Enza Mozzillo, Roberta Pajno, Emanuela Scarano, Maria E. Street, Malgorzata Wasniewska, Sarah Bocchini, Carmen Bucolo, Raffaele Buganza, Mariangela Chiarito, Domenico Corica, Francesca Di Candia, Roberta Francavilla, Nadia Fratangeli, Nicola Improda, Letteria A. Morabito, Chiara Mozzato, Virginia Rossi, Concetta Schiavariello, Giovanni Farello, Lorenzo Iughetti, Vincenzo Salpietro, Alessandro Salvatoni, Mara Giordano, Graziano Grugni, Maurizio Delvecchio

**Affiliations:** ^1^ Department of Medical and Surgical Sciences for Mother, Children and Adults, Pediatric Unit, University of Modena and Reggio Emilia, Modena, Italy; ^2^ Department of Pediatrics, University of L’Aquila, L’Aquila, Italy; ^3^ Department of Medical and Surgical Sciences for Mother, Children and Adults, Post-Graduate School of Pediatrics, University of Modena and Reggio Emilia, Modena, Italy; ^4^ Department of Internal Medicine and Therapeutics, University of Pavia, Pavia, Italy; ^5^ Pediatric Department, Buzzi Children’s Hospital, Milano, Italy; ^6^ Center for Rare Diseases and Congenital Defects, Fondazione Policlinico Universitario A. Gemelli, IRCCS, Rome, Italy; ^7^ Pediatric Endocrinology, Regina Margherita Children Hospital – Department of Public Health and Pediatric Sciences, University of Torino, Torino, Italy; ^8^ Pediatric Unit, Department of Precision and Regenerative Medicine and Ionian Area, University of Bari “Aldo Moro”, Bari, Italy; ^9^ Prader Willi Reference Center, Endocrinology and Diabetology Unit, Pediatric University Department, IRCCS Bambino Gesù Children Hospital, Rome, Italy; ^10^ Pediatric Endocrinology Unit, University Hospital of Padova, Padova, Italy; ^11^ Neuro-endocrine Diseases and Obesity Unit, Department of Neurosciences, Santobono-Pausilipon Children’s Hospital, Naples, Italy; ^12^ Department of Translational and Medical Science, Section of Pediatrics, University of Naples Federico II, Naples, Italy; ^13^ Pediatric Unit, IRCCS San Raffaele Institute, Milan, Italy; ^14^ Pediatric Unit, IRCCS Azienda Ospedaliero-Universitaria di Bologna, Bologna, Italy; ^15^ Department of Medicine and Surgery, University of Parma, Parma, Italy; ^16^ Department of Medicine and Surgery, University Hospital of Parma, Parma, Italy; ^17^ Department of Human Pathology of Adulthood and Childhood, University of Messina, Messina, Italy; ^18^ Pediatric Unit, Gaetano Martino University Hospital of Messina, Messina, Italy; ^19^ Division of Auxology, Istituto Auxologico Italiano, Istituto di Ricovero e Cura a Carattere Scientifico (IRCCS), Verbania, Italy; ^20^ Child and Women Health Department, University of Padova, Padova, Italy; ^21^ Department of Clinical Medicine, Public Health, Life and Environmental Sciences, University of L’Aquila, L’Aquila, Italy; ^22^ Department of Biotechnological and Applied Clinical Sciences, University of L’Aquila, L’Aquila, Italy; ^23^ Pediatric Department, Insubria University, Varese, Italy; ^24^ Laboratory of Genetics, Struttura Complessa a Direzione Universitaria (SCDU) Biochimica Clinica, Ospedale Maggiore della Carità, Novara, Italy; ^25^ Department of Health Sciences, University of Piemonte Orientale, Novara, Italy

**Keywords:** Prader-Willi syndrome (PWS), genotype-phenotype correlation, growth hormone (GH), metabolic syndrome, hypogonadism, bone metabolism, type 2 diabetes, thyroid

## Abstract

Prader-Willi syndrome (PWS) is a complex genetic disorder caused by three different types of molecular genetic abnormalities. The most common defect is a deletion on the paternal 15q11-q13 chromosome, which is seen in about 60% of individuals. The next most common abnormality is maternal disomy 15, found in around 35% of cases, and a defect in the imprinting center that controls the activity of certain genes on chromosome 15, seen in 1-3% of cases. Individuals with PWS typically experience issues with the hypothalamic-pituitary axis, leading to excessive hunger (hyperphagia), severe obesity, various endocrine disorders, and intellectual disability. Differences in physical and behavioral characteristics between patients with PWS due to deletion versus those with maternal disomy are discussed in literature. Patients with maternal disomy tend to have more frequent neurodevelopmental problems, such as autistic traits and behavioral issues, and generally have higher IQ levels compared to those with deletion of the critical PWS region. This has led us to review the pertinent literature to investigate the possibility of establishing connections between the genetic abnormalities and the endocrine disorders experienced by PWS patients, in order to develop more targeted diagnostic and treatment protocols. In this review, we will review the current state of clinical studies focusing on endocrine disorders in individuals with PWS patients, with a specific focus on the various genetic causes. We will look at topics such as neonatal anthropometry, thyroid issues, adrenal problems, hypogonadism, bone metabolism abnormalities, metabolic syndrome resulting from severe obesity caused by hyperphagia, deficiencies in the GH/IGF-1 axis, and the corresponding responses to treatment.

## 1 Introduction

Prader-Willi syndrome (PWS; MIM: 176270) is a rare and complex progressive disorder that affects multiple body systems, and is recognized as one of the most common causes of genetic obesity. It occurs in approximately 1 in 20,000-30,000 live births (1, 2), with an equal number of males and females affected.

### 1.1 Genetic background

**Figure 1:**
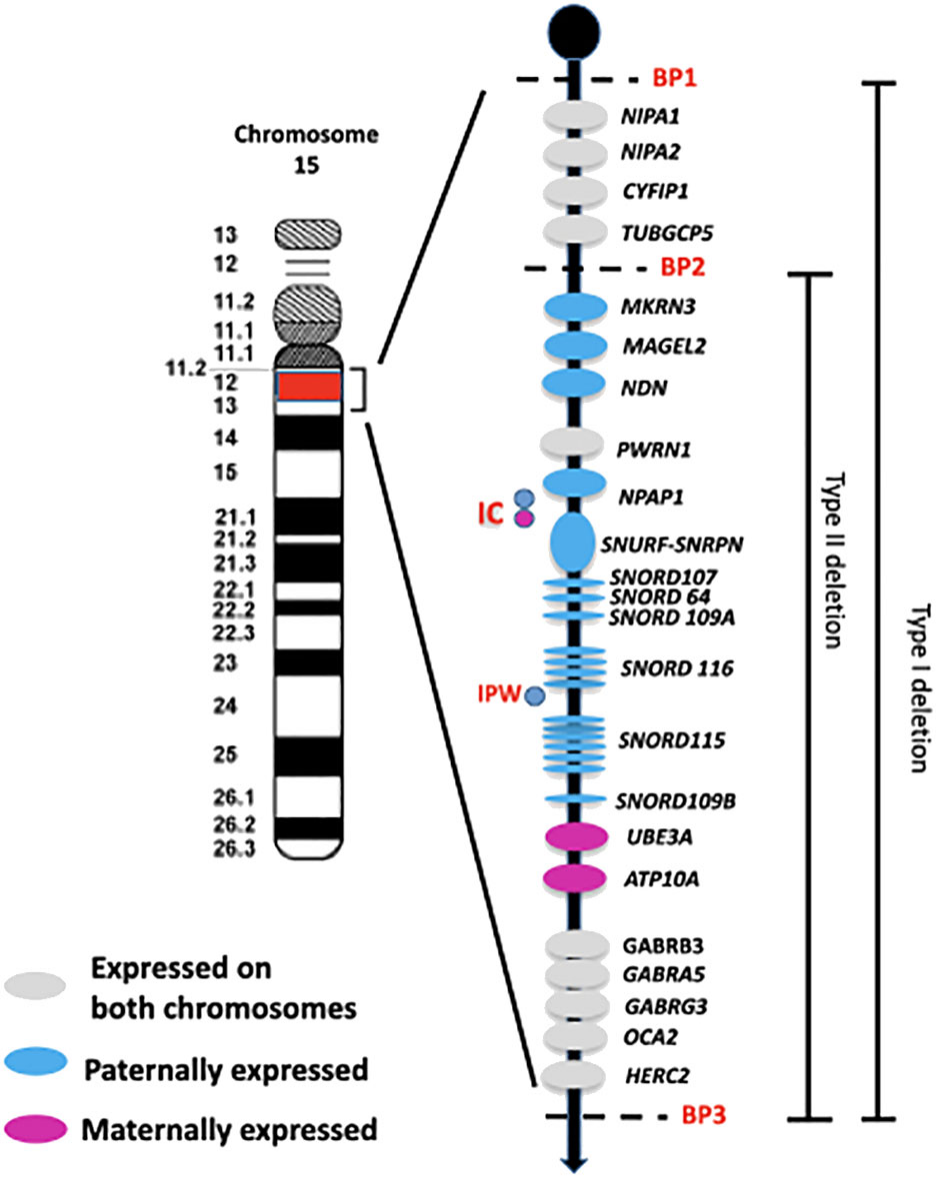
Schematic overview of the imprinted region 15q11q13. The paternally and maternally expressed genes are reported. The typical larger 15q11-q13 type I deletion involving breakpoints BP1 and BP3 and the typical smaller 15q1-q13 type II deletion involving breakpoints BP2 and BP3 are illustrated. IC: imprinting center controlling the activity of imprinted genes in the 15q11-q13 region. IPW: imprinted in Prader Willi syndrome. BP common breakpoint.

Approximately 60% of patients with PWS have a deletion on the paternal chromosome 15q (del15q), located between common breakpoints (BPs – two proximal sites known as BP1 and BP2, and one distal site named BP3). There are two main classes of deletions: type I, which accounts for 40% of cases and is about 6 Mb in size spanning BP1-BP3, and type II, which makes up 60% of cases and spans 5.3 Mb between BP2 and BP3 (3) (**Figure 1**). In addition, around 8% of individuals with a deletion have a unique or atypical deletion size (i.e., not type I or II) due to various causes, such as unbalanced translocations (4).

Maternal uniparental disomy (mUPD), where both copies of chromosome 15 come from the mother, is responsible for approximately 35% of PWS cases and results from errors during meiosis. There are three types of mUPD: i) isodisomy, caused by errors in meiosis II leading to non-disjunction of sister chromatids followed by trisomy rescue; ii) heterodisomy, caused by errors in meiosis I with non-disjunction of homologous chromosomes and no crossing over; and iii) mixed UPD, related to errors in meiosis I with crossover events (3).

Finally, 1-3% of individuals with PWS, who have apparently normal chromosomes, have imprinting defects. PWS was the first human disorder to be linked to genomic imprinting (5). It occurs when genes inherited from the father on chromosome 15q11.2-q13 are not expressed, due to various genomic mechanisms (3). Most genes in this region are subject to genomic imprinting, meaning only the father’s alleles are active while the mother’s alleles are silenced through methylation.

This means that either the paternal chromosome has a maternal imprint-switch or the maternal chromosome has a paternal imprinting. This incorrect epigenotype is a result of a dysregulation in the imprinting mechanism in the parent’s germ line, often caused by microdeletions in a critical region known as the PWS-imprinting center (6), a critical region involved in the regulation of the epigenetic modifications at this locus. In families affected by PWS due to a deletion in the imprinting center, the smallest overlapping region of deletion (PWS-SRO) is 4.3 kb and includes exon 1 of the SNRPN gene (7).

The region between BP1 and BP2 on chromosome 15q11.2-q13 contains four non-imprinted genes: NIPA1, NIPA2, CYF1P1, and TUBGCP5 (**Figure 1**). A microdeletion of this 500 kb region does not cause PWS, but instead leads to a range of clinical manifestations, such as neurodevelopmental disorders that can include intellectual disability, speech impairment, and behavioral disorders. This collection of symptoms is collectively known as ‘Burnside-Butler syndrome’ (8), although not all individuals with this deletion show symptoms.

The PWS critical region is located between BP2-BP3 and contains genes that are crucially involved in growth processes, neurodevelopmental and hormonal regulation. These genes include *MKRN3*, *MAGEL2*, *NDN*, and the bicistronic *SNURF-SNRPN*, as well as C15orf2/NPAP1, a gene that is expressed from both alleles in the testis but only from the paternal allele in the brain (9).

The MKRN3 gene encodes a protein that belongs to a family of zinc finger proteins that act as an E3 ubiquitin ligase. MKRN3 seems to be involved in regulating the hypothalamic-pituitary-gonadal axis by inhibiting the release of gonadotropin-releasing hormone (GnRH) and contributing to the control of puberty onset (10). Pathogenic variants in MKRN3 can disrupt this regulatory function, leading to conditions such as central precocious puberty (CPP) (11). *MAGEL2* encodes the melanoma antigen L2, a member of the Melanoma Antigen Gene (MAGE) family of ubiquitin ligase regulators. It is highly expressed in the hypothalamus, where it plays a crucial role in the regulation of protein trafficking and recycling, which affect secretory granule biogenesis (12). Inactivating mutations in *MAGEL2* cause Schaaf-Yang syndrome (MIM: 615547), which is characterized by delayed psychomotor development, frequent intellectual disability, hypotonia, feeding difficulties, and various dysmorphic facial features.

The NDN gene encodes necdin, a member of the MAGE protein family that is mainly expressed in the brain. It is involved in the regulation of cell proliferation, differentiation, and survival, and is closely linked to the suppression of postmitotic neuron-specific growth. Additionally, it contributes to the regulation of neural stem cells, neuronal differentiation, and synaptic plasticity (13).

In this region, there is also a cluster of more than 80 C/D box small nucleolar RNA genes (snoRNAs) and several antisense transcripts. Within this cluster, SNORD116 is a cluster of genes that code for small nucleolar RNAs that are highly expressed postnatally in the hypothalamus, and are involved in regulating food intake and energy balance.

The correlation between genotype and phenotype in PWS remains unclear, as none of the genes in the PWS region have been specifically implicated in the syndrome on their own (6, 14). However, it is likely that these genes contribute to different extents to some aspects of the phenotype. Several studies suggest that SNORD116 snoRNA plays a key role in the cardinal PWS phenotype (3). While large deletions are the most common genetic defect in PWS, a few patients have been reported with microdeletions that specifically include the tandem array of 30 copies of *SNORD116*. Despite the function of *SNORD116* remaining elusive, these patients exhibit the main clinical features of PWS (15).

Thanks to advancements in molecular genetic testing, the diagnosis of PWS can now be confirmed very early in the neonatal period. The main genetic abnormalities - del15q, mUPD, and imprinting center defect - can all be identified using Comparative Genome Hybridization (aCGH) in combination with methylation-specific multiplex ligation-dependent probe amplification analysis (MS-MLPA). However, a high-resolution karyotype is still needed to detect cases with translocations or other rare chromosomal rearrangements involving chromosome 15 (16).

### 1.2 Endocrine features

It is important to note that PWS undergoes a complex evolution throughout an individual’s lifespan. Typically, the characteristic clinical presentation becomes more apparent in adulthood (17, 18). In infants, major symptoms include severe muscle weakness, weak crying, lethargy, difficulty feeding leading to failure to thrive, as well as cryptorchidism in males and underdeveloped labia in females (19). During childhood, individuals with PWS display hyperphagia, a strong desire for food, and develop severe obesity along with various related complications such as impaired glucose metabolism, type 2 diabetes mellitus (T2DM), fatty liver, gallstones, dyslipidemia, hypertension, metabolic syndrome, and cardiovascular disease unless food intake is controlled (16, 20).

Hyperphagia in PWS is still not fully understood, and controlling appetite remains a challenge in managing these patients. However, the description of the six nutritional phases in PWS has significantly improved our understanding of the natural history of the syndrome (21). Dysmorphic features such as a characteristic facial appearance, small hands and feet, and narrow hands with a straight ulnar border are common (5). Other manifestations include distinct behavioral profiles associated with intellectual disability, such as cognitive rigidity, behavioral outbursts, severe anxiety, skin-picking, and an increased risk of psychosis. Additionally, individuals with PWS may experience scoliosis and/or kyphosis, sleep abnormalities including both central and obstructive apnea, and multiple endocrine abnormalities (22). Particularly, endocrinopathies described in PWS include short stature and growth hormone deficiency (GHD), hypothyroidism, central adrenal insufficiency (CAI), isolated precocious pubarche, and hypogonadotropic and hypergonadotropic hypogonadism (16).

Clinical manifestations related to PWS appear to involve a complex dysregulation of the hypothalamus, affecting both the adeno- and neurohypophysis (23). The exact cause of this dysregulation is not well understood. It is important to recognize that the symptoms of PWS can vary in severity among individuals. Additionally, no specific genotype of PWS is linked to exclusive symptoms, but a diverse range of clinical phenotypes has been observed in patients with the main subtypes (del15q or mUPD) (3, 6).

## 2 Aim and search methods

Genotype-phenotype correlation is a relationship that examines how the presence of a clinical trait corresponds with a group of similar mutations. However, this correlation is reliant on accurate phenotyping. Studying genotype-phenotype correlations can offer insights into disease development, progression, severity, and activity, ultimately aiding clinicians in tailoring treatment plans and follow-up care. Pathways from genotype to phenotype in PWS are difficult to explain. So far, no genotype has been linked to any exclusive symptoms. The aim of this literature review is to describe and discuss the endocrine and metabolic issues underlying PWS, emphasizing potential genotype-phenotype correlations to assist healthcare professionals in their practice and to guide future research endeavors, in order to provide some benefit to people with the syndrome and their families.

Manuscripts were included after conducting a search on PubMed, Directory of Open Access Journals and Google Scholar using the keyword “Prader-Willi syndrome” and the keyword for each specific section (for instance “thyroid” or “growth hormone” etc.). Only manuscripts written in English were considered. We excluded review paper, and non-original research articles. Furthermore, additional papers were included if they were known to the authors of this review, even if they were not retrieved by the search. Finally, the references of relevant articles were screened.

## 3 Neonatal anthropometry

Delayed diagnosis of PWS may be partially due to a lack of timely recognition of perinatal and/or neonatal features. In fact, during the perinatal and neonatal period, PWS is often associated with a higher prevalence of labor induction, breech presentation, cesarean delivery, preterm or post-term delivery, low birthweight, and small for gestational age (SGA) newborns compared to the general pediatric population (19, 24). Pregnancy-related complications, such as polyhydramnios from reduced fetal swallowing causing uterine distension (19) and/or placental abnormalities due to the lack of beneficial influence of paternal genes (25, 26), have been suggested to explain preterm labor and SGA, respectively. Birth weight and weight-for-length of newborns with PWS are reduced by approximately 500 grams (27). Regarding birth length, the results from the literature are still inconclusive, with some studies reporting values comparable to healthy controls (26, 28), and others reporting values 1-2 cm shorter than normal (24, 27). Occipito-frontal circumference was found to be within normal ranges compared to the general population at birth (24, 27), as well as over the first year of life (29). A recent nationwide multicenter study provided growth charts of birth weight and length for gestational age specific for PWS (27), which may serve as a useful tool in supporting the suspicion of PWS in newborns.

To date, only a few studies have examined potential links between perinatal characteristics and the specific genotype of PWS, resulting in conflicting findings. The differences in the prevalence of preterm births among different genotypes remain uncertain, as some researches have reported similar rates between mUPD and del15q (24, 26, 30, 31), while others have found a higher prevalence in mUPD individuals (32, 33) or, conversely, in those carrying del15q (19). Additionally, mUPD has been linked to a greater likelihood of post-term births (32).

Most studies have not identified differences in birth weight among specific genotype groups (26, 27, 31, 34). However, Dudley et al. discovered lower birth weight only in the del15q group, irrespective of gender (28). On the other hand, the prevalence of SGA was significantly higher in mUPD carriers compared to patients with del15q or imprinting center defects, with a prevalence rate of 31%, roughly twice as high as in the other two genotype groups (24). Furthermore, several studies have indicated a correlation between parental weight (28) or maternal age (24, 26) and birth weight specifically in PWS patients with mUPD or imprinting center defects.

Regarding birth length, a large cohort study (27) found a significant decrease only in females with del15q compared to those with mUPD, with no clear genotype-phenotype correlation observed for male neonatal size. These results contradict those reported by Whittington et al. (26), who found lower birth length values only in patients with mUPD or imprinting center defects, who were also shorter than patients with the del15q.

## 4 Thyroid function

Thyroid function in PWS has been extensively studied, and, like other endocrine disorders, its cause is believed to be centrally located in origin due to a dysregulated hypothalamic-pituitary-thyroid (HPT) axis.

In PWS, central hypothyroidism (CH) is the most common thyroid function abnormality, with a prevalence ranging from 2% to 72.2% globally (35, 36) and varying age of onset (**Supplementary Table 1**). Screening for hypothyroidism in newborns with PWS often produces negative results, as their levels of TSH and total T4 are typically within the normal range and comparable to those of healthy individuals (37). However, there have been a few cases of congenital hypothyroidism in PWS newborns due to an ectopic thyroid gland (38, 39).

Congenital hypothyroidism appears to be more common in infants and toddlers with PWS, with the highest incidence in the 1 to 3-year-old group, gradually decreasing over time (40), likely due to a temporary dysfunction of the hypothalamic-pituitary-thyroid axis (41, 42). During this phase, treatment with levothyroxine may be necessary if there is a simultaneous decrease in free T4 (fT4) and free T3 (fT3) levels, or an increase in TSH levels, to prevent significant impacts on intellectual development (43, 44). In later stages of life, thyroid function may normalize, even in individuals who were previously treated with levothyroxine (44). It is recommended that adults with PWS undergo regular and periodic screening of fT4 and TSH levels, as untreated hypothyroidism can have adverse effects on metabolic rate, body mass index, and cardiovascular health (45).

The influence of sex on thyroid status and whether genotype could be associated with a different pattern of thyroid function still remains unclear. Regarding genotype, the prevalence of CH in children with PWS was not significantly different among the paternal deletion, mUPD, and unclassified groups (40). Similarly, no relationship between hypothyroidism and genotype was found in a cohort of 122 adults with PWS (45). Recent studies have shown that there are no significant differences in the prevalence of CH between the sexes (42), although previous works observed that CH was more common in males with PWS (41).

The relationship between thyroid function and recombinant human (rhGH) therapy, particularly the influence of rhGH on serum thyroid hormone levels and increased conversion of thyroxine (T4) to triiodothyronine (T3), has been evaluated in some studies (41, 46). RhGH therapy does not seem to increase the prevalence of different forms of hypothyroidism in PWS (41, 46), but it is important to evaluate thyroid hormones before, during, and after rhGH treatment to confirm euthyroidism and prevent the possible risks of previously unmasked and/or untreated hypothyroidism.

The autoimmune cause of impaired thyroid function has never been described or studied in patients with PWS. Thyroid autoantibodies are rarely measured in PWS patients, likely due to the well-known central origin of hypothyroidism. Only one study, which evaluated 21 PWS children, reported a case of mild positivity for anti-thyroid peroxidase antibodies (39). However, the possibility of autoimmune thyroid involvement could be considered, given the recently proposed autoimmune cause of pituitary dysfunction associated with PWS (47, 48). Further research is needed to clarify this issue.

Regarding hyperthyroidism, a single case with an unclear origin treated with thiamazole was reported in a group of adult patients with PWS (45). It is important to regularly monitor the thyroid function of PWS patients at different stages of life, taking into account factors such as levothyroxine treatment, rhGH therapy, and the emergence of new symptoms of hypothyroidism.

Based on the data available in the literature, there does not appear to be a clear correlation between genotype and thyroid dysfunction in individuals with PWS. Yang et al. (42) found that infants with PWS caused by mUPD had higher TSH levels compared to those with PWS caused by a paternal 15q11-q13 deletion, even though the TSH levels were still within the normal range. Additionally, there were no differences in fT3 and fT4 levels between these two groups. Conversely, in adults with PWS caused by a 15q11-q13 deletion, TSH levels within the normal range were higher than in PWS individuals with mUPD. Again, no differences in fT3 and fT4 levels were found between the groups (45). Therefore, further evaluation will be necessary to determine if there is a possible correlation between genotype and the different patterns of thyroid dysfunction.

## 5 Adrenal gland function

Disturbances in the hypothalamus-pituitary-adrenal (HPA) axis, with an inadequate response during stressful conditions like infections or episodes of dehydration, have been suggested as potential causes of unexpected and unexplained deaths in PWS. This theory is supported by the discovery of adrenal atrophy upon autopsy (49) and reduced cell numbers in the hypothalamic paraventricular nuclei (50).

CAI in PWS is not well understood, as studies have reported varying results (ranging from 0% to 60%) using different dynamic tests, such as the insulin tolerance test (ITT), metyrapone test (MT), glucagon test (GT), and the currently most used low-dose and standard dose Synacthen stimulation test (LDSST and SDSST) (**Table 1**) (51–62).

**Table 1:** Summary of the studies with stimulation tests for central adrenal insufficiency in PWS.

Author, year	Subjects involved	Methods (test, normal values)	Diagnosis of CAI	Genotype-phenotype correlation
De Lind Van Wijngaarden, 2008 (51)	25 children	MT (ACTH ≥ 33 pmol/l)	15 (60%)	No significant difference in genotype between patients with and without CAI
Corrias, 2012 (52)	84 children	LDSST, followed by confirmatory SDSST (cortisol > 500 nmol/l, for SDSST also an increase in cortisol of ≥ 250 nmol/l)	4 (4.8%)	In patients with del15q the cortisol peak was significantly lower than in mUPD cases; mUPD genetic subclass resulted a predictor of peak response
Grugni, 2013 (53)	53 adults	LDSST, followed by confirmatory SDSST (cortisol > 500 nmol/l)	4 (7.5%)	Genetic abnormality was not predictive of CAI
Obrynba, 2018 (54)	21 patients (4-53 years)	LDSST (cortisol ≥ 427.6 nmol/l) followed by MT (11-deoxycortisol ≥ 200 nmol/l)	0	No statistically significant difference between del15q and mUPD in LDSST results
Oto, 2018 (55)	36 children	ITT (cortisol > 18.1 µg/dl - 499.3 nmol/l- or increase in cortisol by ≥ 9.1 µg/dl - 251 nmol/l)	0	In PWS subjects with del15q peak levels of cortisol after ITT were significantly delayed compared to mUPD patients

CAI, central adrenal insufficiency; del15q, deletion of chromosome 15; ITT, insulin tolerance test; LDSST, low-dose and standard dose Synacthen stimulation test; MT, metyrapone test; mUPD, maternal uniparental disomy; SDSST, standard dose Synacthen stimulation test.

Based on early data that show a high prevalence of CAI (56, 56), some authors have suggested that all patients with PWS should be treated empirically with hydrocortisone during stressful situations unless CAI has been ruled out. The high prevalence reported in these studies, however, could be related to the use of ACTH levels for the diagnosis of CAI, rather than 11-deoxycortisol, and the high cut-off used (33 pmol/l) (54, 57, 61). More recent studies, using 11-deoxycortisol or a lower cut-off of ACTH after MT or other dynamic tests, reported a much lower prevalence (52–55, 57–62). Based on this low CAI prevalence, several authors advised against routine administration of hydrocortisone during psychological stress, illness, or surgery (61) to avoid excessive treatment. Additionally, reports of adrenal crisis during surgery are currently only anecdotal (63). Other reports by Oto et al. (55) and Grootjen et al. (62) have shown a delayed peak of cortisol after ITT, ACTH and 11-deoxycortisol after MT.

The literature data generally supports the evidence that individuals with PWS in common clinical practice do not typically experience adrenal insufficiency, are not usually treated with daily glucocorticoids, and do not generally experience adrenal crises during surgery or other stressful procedures and events. Currently, there is no consensus among endocrinologists on the best diagnostic approach, management, or need for empiric glucocorticoid treatment during critical illness or before surgery.

Regarding the genotype-phenotype correlation, it is currently not possible to define a clear correlation, as evaluated by a small number of authors so far. de Lind van Wijngaarden et al. (51) found no significant differences in genotype between pediatric patients with and without CAI diagnosed by MT, while Corrias et al. (52) reported a significantly lower cortisol peak after LDSST in children with del15q compared to mUPD, a genetic subclass identified as a predictor of the peak response. However, this result with LDSST was not confirmed by Obrynba et al. in children (54) or by Grugni et al. in adults (53). Oto et al. (55) did indicate a delayed cortisol peak response after ITT in del15q compared to mUPD.

Regarding basal cortisol, children with PWS are generally able to produce adequate levels in daily life, as demonstrated by studies showing normal average morning basal serum cortisol (64–66) or salivary cortisol (51). Lower values of morning basal cortisol, although within the normal range compared to the general population, were reported in a large cohort of PWS children by Angulo et al. (64), but not by Beaulove et al. (60) and Oto et al. (55), nor by Butler et al. in a population of adults and children with PWS compared to a control group with non-syndromic obesity (65). Angulo et al. and Butler et al. (64, 65) found no correlation between morning basal cortisol and specific genetic subtypes. Based on the literature review, primary adrenal insufficiency (PAI) has not been reported in PWS patients.

Another issue regarding adrenal function in PWS is the presence of premature pubarche, reported in the literature with a very variable prevalence, from 14% to 60% (66–73). Siemensma et al. (67) and Gaston et al. (70) found no significant differences in the proportion of patients with premature pubarche in subjects with different genetic subtypes. Increased plasma levels of DHEA-S have been reported in children with PWS compared to the healthy pediatric population, with no associations between DHEA-S levels and BMI or percentage of fat (66–68). Finally, Siemensma et al. (67) showed no significant differences in DHEA-S levels between genetic subtypes in children, while Gaston et al. (70) reported the highest DHEA-S levels in patients with del15q.

## 6 Bone metabolism

Individuals with PWS typically have a bone structure characterized by short stature, low bone mineral density (BMD), and low bone mineral content (BMC) (74), often leading to orthopedic issues such as scoliosis, kyphosis, hip dysplasia, flat feet, and genu valgum (75, 76).

The process of bone formation is continuous throughout life, and the acquisition of bone minerals in early childhood is crucial for achieving optimal BMD levels in late adolescence. Bone health is influenced by the interaction between osteoblasts (bone forming cells, OBs) and osteoclasts (bone resorbing cells, OCs), which are primarily regulated by the Wnt/β-catenin pathway and the receptor activator of nuclear factor-κB (RANK)/RANK ligand (RANKL)/osteoprotegerin (OPG) axis, respectively (74).

In childhood, individuals with PWS often have normal BMD levels when adjusted for their reduced height (77), but during adolescence, they may experience a decrease in total BMD and BMC. As a result, it is estimated that 29% to 44% of adults with PWS may suffer from bone fractures due to the higher prevalence of osteoporosis, reported to be as high as 21% (78).

The factors contributing to bone impairment in individuals with PWS are not fully understood, but possible explanations include loss of function of genes in the q11-q13 region of the paternal copy of chromosome 15, reduced production of sex hormones during puberty, as well as a relative GHD during childhood and adolescence (5). Additionally, low calcium intake, insufficient levels of vitamin D, lack of physical activity, and changes in serum adipokines may also play a role in bone impairment in these individuals (79).

Regarding the genes in the PWS critical domain that are involved in skeletal abnormalities, deletion of Snord116 has been shown to have negative effects specifically on the bone cortical compartment (80). Deletion of *MAGEL2* is linked to Schaaf-Yang syndrome, which is characterized by a unique skeletal phenotype with abnormal BMD due to increased OCs activity and enhanced transformation of OBs into adipocytes (81). *MAGEL2* deletion is also associated with decreased levels of N-oleoyl serine, which has a positive correlation with BMD and OBs activity (82).

Hypogonadism is a common feature in individuals with PWS. This condition is a well-recognized risk factor for osteoporosis, and it is important to start hormone replacement therapy (HRT) in a timely manner to prevent bone loss (78). A cohort study of 22 PWS patients showed a significant improvement in BMD and lean body mass after starting HRT (83). Another study found that individuals with PWS who received HRT had higher BMD compared to those who were not treated (84). Therefore, it is recommended that individuals with PWS start HRT as soon as hypogonadism is identified.

There is limited data on the effects of rhGH treatment on BMD in individuals with PWS. A study by Bakker et al. (85) found that total body BMD and lumbar spine BMD remained stable in prepubertal PWS children during 9 years of GH therapy, but decreased during adolescence due to incomplete pubertal development, emphasizing the importance of prompt replacement therapy. In a group of adult PWS subjects, GH treatment for 2 years did not effectively improve low bone mass (86). However, there are conflicting findings on the effects of GH therapy on BMD in PWS, as some studies have shown that GH administration in PWS adults has a positive effect on bone mineralization and geometry (87). Nakamura et al. (88) reported a low BMD in 61.5% of subjects with PWS, with an improvement in lumbar BMD Z-score after more than 4 years of GH administration. Additionally, a higher incidence of osteoporosis was found among PWS adults who did not receive GH therapy during adolescence (88).

Data on vitamin D levels in individuals with PWS are still limited and inconclusive. Panfili et al. (89) studied a group of pediatric PWS patients and did not find any significant differences between PWS children and the control group, although there was a slight decrease in obese PWS individuals compared to those of normal weight. In contrast, Barrea et al. discovered that adults with PWS had lower levels of vitamin D compared to the control group, regardless of differences in body fat (90).

Brunetti et al. demonstrated the involvement of the Wnt/β-catenin and RANK/RANKL/(OPG) axis in PWS by showing high levels of RANK-L and low levels of OPG in both children and adults with PWS, with a bias towards RANK-L (91). Additionally, sclerostin levels were significantly higher in children and lower in adults with PWS compared to controls, indicating that RANK-L, OPG, and sclerostin may play a crucial role in regulating bone turnover in individuals with PWS.

Recently, irisin, a myokine secreted by the muscle, has sparked great interest due to its involvement in bone, adipose tissue, and brain homeostasis. Specifically, in young mice, irisin injection mimicked the effects of exercise by increasing cortical bone mass and strength (92). Hirsch et al. found higher amounts of salivary irisin in obese individuals with PWS compared to non-obese controls, while plasma levels of irisin did not show significant changes between the two groups (93, 94). Faienza et al. demonstrated that irisin serum levels in PWS patients were similar to those of controls (95), however, both pediatric and adult PWS subjects with del15q had lower irisin levels compared to controls, whereas no difference was observed between PWS subjects with mUPD and healthy controls.

LIGHT/TNFSF14 is a cytokine produced by immune cells that affects both fat and bone metabolism; Faienza et al. showed that serum LIGHT levels were significantly higher in both PWS children and PWS adults than in controls (96). Additionally, LIGHT levels were found to have a negative correlation with DEXA parameters. LIGHT serum levels were influenced by various factors including vitamin D and DXA parameters related to bone and fat quality, indicating the important role of LIGHT as a marker of bone impairment.

**Table 2** summarizes the most important studies about bone metabolism in PWS.

**Table 2:** Summary of the studies about bone metabolism in PWS.

Author, year	Objective	Subjects	Results
Bakker NE, 2015 (85)	To determine effects of long-term GH treatment and puberty on BMD of total body and lumbar spine	77 children with PWS who remained prepubertal during GH treatment for 4 years and 64 children with PWS who received GH treatment for 9 years participated in the study	Total body BMD and lumbar spine BMD remain stable in prepubertal PWS children but decreases during adolescence, due to the incomplete pubertal development
Jørgensen AP, 2013 (86)	To investigate bone mass in a group of adult PWS subjects and study the effects of GH treatment on BMD	46 adults with PWS were randomized to GH or placebo for 12 months, followed by open prospective GH for 24 additional months	No changes in BMD were observed with continuous GH treatment for 24 months
Longhi S, 2015 (87)	To evaluate bone geometry, density and strength in a group of adult obese patients with PWS and to examine the effect on bone of treatment with GH and sex steroids	41 adults with PWS treated with GH and 46 healthy controls	GH treatment improves bone geometry but not bone density. Bone strength was significantly reduced in PWS patients who did not receive GH and had been treated with sex steroids.
Nakamura Y, 2014 (97)	To investigate BMD in PWS patients and to verify the efficacy of and scoliosis deterioration with GH administration for osteoporosis	148 PWS (141 treated with GH) patients who underwent lumbar spine BMD testing	61.5% of subjects had low BMD; GH administration significantly improved the lumbar BMD
Panfili FM, 2023 (89)	To analyze 25OHD levels in pediatric PWS patients in comparison with a control group	192 children and adolescents with PWS and 192 healthy controls	No statistically significant differences in 25OHD levels were observed between the PWS population and the controls; a slightly decrease was observed in obese than normal weight PWS
Barrea L, 2020 (90)	To investigate 25OHD levels and the dietary vitamin D intake in PWS adults	15 PWS adults and 15 control subjects	PWS had lower 25OHD levels than in the control group, regardless of body fat differences
Brunetti G, 2018 (91)	To test the hypothesis that the levels of bone remodeling mediators (RANKL, OPG, sclerostin, DKK-1 serum levels, and bone metabolism markers) may be altered in PWS subjects	12 PWS children, 14 PWS adults and 31 healthy controls	High RANKL and low OPG serum levels were found both in children and adults; sclerostin serum levels were significantly higher in children and lower in adults than controls
Faienza MF, 2021 (95)	to determine circulating levels of irisin in children and adult PWS patients	78 subjects with PWS (26 children and 52 adults) and 80 healthy controls (26 children and 54 adults)	Irisin serum levels in PWS patients did not differ when compared with controls but both pediatric and adult PWS with del15q displayed lower irisin levels than controls while no difference was observed between PWS subjects with mUPD and healthy controls; in pediatric PWS the vitamin D levels affected irisin serum concentration
Faienza MF, 2023 (96)	To evaluate LIGHT serum levels and to identify correlations with parameters of bone and fat metabolism	8 children and 52 adult PWS patients compared to age and sex-matched controls	Serum LIGHT levels were significantly higher in both PWS children and in PWS adults than controls; LIGHT was also negatively correlated with DEXA

BMD, bone mineral density; del15q, deletion of chromosome 15; DEXA, dual-energy x-ray absorptiometry; GH, growth hormone; mUPD, maternal uniparental disomy; RANK, receptor activator of nuclear factor-κB; RANKL, RANK ligand; OPG, osteoprotegerin; 25OHD, 25-hydroxy vitamin D.

## 7 Growth hormone - insulin-like growth hormone -1 axis and growth

Impaired growth is a common feature of PWS, attributed to a lack of GH/IGF-1 and a lack of pubertal growth spurt. Infants with PWS also show other clinical signs of GHD, such as low muscle tone, small hands and feet, increased body fat percentage, and reduced lean mass.

GHD is documented in 40-100% of children with PWS, although the severity can vary from mild to severe (98–100). Without rhGH replacement therapy, the average final height for men is reported to be 155-160 cm, and for women, it is 145-150 cm (101).

The cause of impaired GH secretion in PWS is still being debated, mainly because early childhood-onset obesity is so common in this population. It is widely recognized that obese individuals who are otherwise healthy tend to have lower GH secretion compared to lean individuals. Obesity typically leads to changes in the IGF system, resulting in lower IGF-1 availability. However, most studies show that the total measured circulating IGF-1 levels in obese individuals are normal or even elevated (102). In individuals with PWS, IGF-1 levels are often reported to be low in many studies, regardless of BMI, indicating a GHD. Additionally, levels of IGFBP-3, a protein that binds to IGF-1, are also found to be low in PWS (99, 103, 104). Unfortunately, research on the IGF system in PWS has been limited overall.

The GH response to growth hormone-releasing hormone (GHRH) stimulation test with simultaneous administration of pyridostigmine in obese children showed increased GH levels, attributed to the reduced induced somatostatinergic tone. Meanwhile, children with PWS still exhibited lower GH response and reduced serum IGF-1 concentrations, confirming a genuine GHD (105). GHD is a progressive process in individuals with PWS. According to Cohen et al., stimulated levels of GH in infants with PWS were higher compared to older children or adults, suggesting that younger children may have a normal GH pituitary reserve (106). Additionally, some studies have indicated that in young children with PWS, standard stimulation tests (clonidine and arginine) result in low GH peaks in most patients, whereas combined stimulation tests (GHRH+arginine or pyridostigmine) show normal results in the majority of PWS tested children. This suggests that very young PWS children may have impaired hypothalamic GHRH secretion with a normal GH pituitary reserve, which could diminish as they grow older, possibly resulting in insufficient levels of GH (107). Therefore, the efficacy of standard GH stimulation tests as an indicator of GH status in younger individuals with PWS is still a topic of debate. Additionally, it has been demonstrated that, using the potentiated GHRH+arginine stimulation test, GH response is greater in children than in adults (108).

Reduced GH bioactivity has also been shown in children and adolescents with PWS. This should be taken into account in the small number of affected individuals with PWS and growth failure who exhibit normal growth hormone responses after standard stimulation tests (109). Additionally, low growth hormone secretion has been found in 24-hour frequent blood sampling studies, which is consistent with neurosecretory GHD based on current knowledge (108).

Furthermore, impaired GH secretion in these individuals is linked to a reduction in visceral fat and elevated ghrelin concentrations relative to the degree of obesity (110). Ghrelin, a stomach-derived hormone that stimulates GH secretion, can be increased in individuals with PWS before the onset of obesity, which is a distinct feature not typically seen in common obesity or other genetic obesities (111, 112). However, due to the various forms of ghrelin and their effects, it is currently challenging to establish clear relationships with GH secretion in PWS (113).

During the transition phase, around 20% of individuals with PWS displayed a GHD status based on BMI-dependent criteria (114), while GHD was found in 8-38% of adult patients (115). Some genotype-phenotype correlations have been identified in relation to GH secretion (116), but there is a lack of literature on the changes in the IGF system (**Table 3**). It is worth noting that patients with mUPD are more likely to have GHD compared to those with deletions (80% versus 25%) (100, 107–109). No significant differences have been observed between individuals with type I and type II deletions (98, 107, 108, 117). Additionally, only those with paternal deletions show a correlation between GH response to stimulation and BMI, which is not seen in subjects with mUPD (108). Patients with paternal gene microdeletions also exhibit better GH responses to stimulation than those with mUPD, who experience delayed GH peaks after stimulation as a distinctive feature. Interestingly, children with del15q11-q13 are longer at birth than those with mUPD, and the latter are shorter than PWS patients without deletions (105). Deletions in PWS, particularly those affecting genes involved in neural development, brain function, infertility, and circadian rhythm, such as SNORD116, can result in proconvertase 1 deficiency leading to GHD, short stature, hypogonadism, hyperghrelinemia, relative hypoinsulinemia, and T2DM. Therefore, special attention should be paid to these children for early replacement treatment consideration (100).

**Table 3:** Observed genotype-phenotype correlations in Prader Willi Syndrome related with GH secretion.

Author, year	GH secretion in PWS	Genotype-Phenotype correlations
Di Giorgio G, 2014; Marostica E, 2013; Alves C, 2020; Grugni G, 2011 (100, 107–109)	Correlation between different PWS genotypes and incidence of GHD	Patients with mUPD have a higher incidence of GHD than those with deletion while no differences have been reported between individuals with both type I and type II deletions
Marostica E, 2013 (108)	Correlation between GHD and BMI in PWS genotypes	A correlation between the GH response to stimulation and BMI has been demonstrated only for individuals with paternal deletion
Di Giorgio G, 2014 (107)	Response to stimulation tests differs in different PWS genotypes	Patients with paternal gene microdeletions present better GH responses to stimulation tests with respect to PWS patients with mUPD
Grugni G, 2021 (114)	Prevalence of GHD in PWS during the transition phase	Patients with del15q had higher GH peak response to GHRH+arginine compared with subjects with mUPD
Grugni G, 2011 (117)	Comparison of stimulated GH response in adults with PWS due to different genetic subtypes	Subjects with mUPD had a lower mean peak GH response and integrated GH secretion than those observed in patients with del15q

BMI, body mass index; del15q, deletion of chromosome 15; GH, growth hormone; GHD, growth hormone deficiency; GHRH, growth hormone-releasing hormone; mUPD, maternal uniparental disomy.

In both the transition phase and in adult patients, the GH peak response to GHRH+arginine was significantly higher in patients with del15q than in those with mUPD (114, 117).

## 8 GH therapy

GH therapy in children with PWS was initially approved by the Food and Drug Administration in 2000 and by the European Medicines Agency in 2001. The Consensus Guidelines of the Growth Hormone Research Society in 2013 recommended GH therapy for all children with genetically confirmed PWS, in addition to dietary, lifestyle, and environmental interventions (118).

The goals of GH therapy in PWS go beyond simply improving growth (99), as it has a positive impact on various clinical aspects and the quality of life of affected individuals. GH therapy results in improved body composition, with a decrease in fat mass and an increase in lean mass (119, 120). It also has a positive effect on muscle strength, motor function (121, 122), and bone mineral density (123). Furthermore, improvements in neurological and behavioral impairments, such as achieving motor milestones earlier and developing adaptive skills, have been reported (124).

In adulthood, GH treatment has been shown to have beneficial effects on lean and fat mass, muscle performance, and quality of life (116). The positive effects on body composition and fat distribution are maintained for an average of 17 years, regardless of GH secretory status (125). However, it is necessary to confirm the presence of GHD after reaching final height before initiating GH therapy (118).

According to previous expert opinions (118, 119), in pediatric age, it is not necessary to assess GH/IGF-1 function before starting GH treatment. However, some contraindications to GH therapy are present in subjects with PWS, such as severe obesity or severe respiratory impairment, acute critical illness, uncontrolled diabetes, active cancer, and active psychosis (118). There is no consensus on the age at which to start therapy, but there is agreement on starting before the onset of obesity, which often occurs at around 2 years of age (118). Some studies have shown a better benefit in starting therapy in the first year of life, between 4 and 6 months (122, 124) or even at 3 months (118). The recommended dose is 1.0 mg/m2 per day, achieved within approximately 3-6 months of starting treatment (118). This dose is higher than that usually used in congenital GHD, but lower doses showed a reduced effect on body composition (126, 127). If body surface area is not used for dose calculation, it is recommended to use a nonobese weight for height as a reference in obese patients (118). PWS patients seem to be particularly sensitive to GH, as at standard doses they often present IGF-1 levels above the normal limits, in particular in individuals with PWS due to mUPD (128); special care must be taken in biochemical monitoring for a possible association between IGF-1 values and the occurrence of adverse effects (129–131). Therefore, it is recommended that IGF-1 levels remain within the upper normal range (+1 to +2 SDS) for age-matched normal children during GH therapy, and it is indicated in infants and children with PWS to start therapy with a daily dose of 0.5 mg/m2 per day to minimize side effects, with subsequent adjustments toward 1.0 mg/m2 per day (117). Scheermeyer et al. reported that treating infants with a low dose of GH (4.5 mg/m2/week) leads to a normalization of IGF-1 and height SDS within the first year of therapy with minimal risk of side effects (132). However, no relationship between malignancies and GH treatment has been reported in a large cohort of patients with PWS (133).

A recent study has shown that the genetic diagnosis of PWS and the start of GH therapy occur earlier in patients with a del15q than in those with mUPD and an imprinting center defect (ID) (31). According to the study, patients with del15q are typically diagnosed in the second year of life, while other PWS patients are diagnosed in the fourth year of life. Additionally, GH therapy is initiated at an average age of 4.24 years in del15q patients, 7.3 years in mUPD 15, and 6.42 years in those with mUPD/ID. Although mean IGF-1 values before GH treatment were within the reference range for age and sex, del15q patients had significantly higher IGF-1 levels compared to mUPD/ID (-0.83 ± 0.46 SDS vs -1.03 ± 0.43 SDS), despite no significant differences in height SDS.

Among patients with mUPD, GHD is more common and severe than patients with a del15q (116). However, Oto and colleagues found that there was no significant difference in yearly height improvement between these two groups of patients (134). Nonetheless, when looking at subgroups meeting the criteria for GHD, mUPD patients showed a significantly better response to rhGH treatment in terms of annual growth rate than deletion patients (0.42 ± 0.26 for del15q vs 0.7 ± 0.21 for mUPD; p=0.0044). Variability in response to GH therapy may also be linked to the presence of an exon-3 deletion polymorphism of the GH receptor (d3 allele), which is present in around 50% of the Caucasian population (135). In children with PWS, those who are heterozygous or homozygous for the d3 allele are more sensitive and responsive to GH treatment, leading to a significant increase in growth compared to those who are homozygous for the full-length GHR allele (136). Similar outcomes are observed in non-PWS children undergoing GH therapy (135).

According to literature, adult patients with del15q show higher body weight and BMI compared to those with mUPD. However, both groups have similar proportions of fat mass, adipocyte volume, and insulin resistance markers (137). These findings suggest that growth hormone therapy during childhood and/or adolescence may lead to a better metabolic profile in adults with PWS who have del15q, but not in those with mUPD, thus lessening the phenotypic impact of the deletion.

In terms of intellectual disability, Butler et al. analyzed cognitive skills and proposed that rhGH therapy could help prevent cognitive decline in individuals with PWS, especially those with mUPD (138).

Differences between mUPD and del15q have also been documented in relation to psychiatric issues: individuals with del15q exhibit higher levels of aggression compared to those with mUPD (p=0.007) (139), are more likely to engage in skin picking (p=0.008) (139) and are more prone to developing compulsions and self-injury (140, 141). On the other hand, mUPD 15 is associated with an increased risk of anxiety disorders (p=0.04) and psychoses (142). Moreover, GH therapy has shown a significant link to the development of anxiety and delusions (2.7 times increased association with anxiety, p=0.05; 14 times increased association with delusions, p=0.03). Specifically, GH therapy poses a higher risk of anxiety in individuals with mUPD (3.25-fold increase) compared to those with del15q (2.73-fold increase), regardless of treatment duration (139).

In relation to orthopedic issues, a higher incidence of scoliosis has been reported among individuals with PWS who have received rhGH treatment and have a deletion (p=0.011). On the other hand, scoliosis is more common in patients with mUPD who have not undergone therapy (p=0.039) (143). However, data from the literature do not show any significant connection between scoliosis and rhGH treatment (144). The same authors also noted that kyphosis occurs in similar proportions in individuals with a deletion and mUPD undergoing rhGH therapy, but among untreated patients, it is more prevalent in those with a deletion rather than mUPD (p=0.001). Additionally, they found differing associations between genotype and phenotype in individuals with a deletion receiving rhGH treatment compared to those with mUPD, particularly in terms of having a flat back of the head (p=0.002) and abdominal stretch marks (p=0.006), which were more common in the former group. They also observed that ocular signs, such as eyes that slant downwards and strabismus, were more frequent in individuals with mUPD receiving growth hormone therapy (p=0.006 and p=0.011).

## 9 Obesity

The clinical symptoms of PWS can vary depending on age. In later childhood, patients can become severely obese unless their food intake is closely monitored by family and caregivers. Individuals with PWS often experience insatiable hunger, known as hyperphagia, and other compulsive behaviors, typically developing between the ages of 2 and 5 years (21, 145, 146). While the exact cause of hyperphagia in PWS is not fully understood, it is believed to involve abnormalities in hypothalamic satiety pathways. This may include hypothalamic malformations or related neurochemical mechanisms (147, 148).

However, abnormal eating behaviors in PWS cannot be solely attributed to hypothalamic dysfunction. Functional brain imaging techniques, such as functional magnetic resonance imaging (fMRI) and fluoro-deoxy-glucose positron emission tomography (FDG-PET), have shown that the orbitofrontal cortex (OFC) is activated when individuals consume food until they feel full. Signals related to satiety and visceral cues within the OFC significantly influence food perception, resulting in a response that reflects the food’s reward value or appeal. Individuals with PWS have been found to have altered OFC responses to satiety in functional brain imaging studies (149). The olfactory system also plays a crucial role in nutrition and social behavior, with connections to the endocrine regulation of energy balance (150). It can trigger a specific appetite for relevant foods, known as sensory-specific appetite, helping us detect and respond to food in our environment. A study involving adults with PWS (150) revealed abnormal brain reward system activity, particularly at the right amygdala level, in response to food odors. This heightened amygdala activity corresponds to the rapid and craving-inducing reaction to the odor, aligning with the concept of food-related addictive behaviors. Additionally, this increased activity is linked to the severity of hyperphagia, suggesting that changes in the brain circuits processing food odors may contribute to this phenomenon in PWS (150). Individuals with PWS have elevated levels of ghrelin and oxytocin, both of which are involved in addictive behaviors. These hormones not only impact the hypothalamus and limbic system but also influence the olfactory bulb, affecting sensory processing. Elevated fasting levels of ghrelin, hyperadiponectinemia, hypoinsulinemia, and increased ghrelin/PYY values compared to obese controls have been described (151). Ghrelin abnormalities may contribute to hyperphagia and food-related thoughts and cravings in PWS, while oxytocin dysfunction may affect emotional processing and appetite regulation (150).

None of the genetic defects associated with PWS are directly linked to specific clinical traits. However, some connections have been found between different molecular classes and clinical features, including eating behavior and obesity.

Several studies have investigated possible differences in compulsive behavior among the three most prevalent genetic subtypes (Type I deletion, Type II deletion, and mUPD). These behaviors often center around food-related problems, like searching for and saving food, acting on impulse, and repeatedly asking for food. These behaviors ultimately play a role in the onset of obesity.

Therefore, it is clear that individuals with mUPD show a lower frequency and severity of compulsions compared to those with deletions. However, the available data on potential differences between the two types of deletions are inconclusive (145). Type I deletions, which affect a greater number of genes, seem to be associated with more severe compulsions than Type II deletions (145). Yet, other studies have found no noticeable distinctions (152, 153).

Dykens et al. (153) conducted a cohort study with eighty-eight individuals (43 males and 45 females) with PWS ranging from 5 to 51 years old. Their research did not find any clear differences between deletion subtypes, but they did notice significant variations within each subtype in terms of the relationship between age and behavior. Specifically, in the Type I group, they found negative correlations between age and behavior, possibly due to non-imprinted genes like CYFIP1. The CYFIP1 gene, which is also involved in other developmental disorders like 15q abnormalities, may contribute to age-related changes in the phenotype of individuals with Type I PWS due to its haploinsufficiency in those cases.

Deficiencies in imprinted genes, such as MKRN3, MAGEL2, and/or NDN, are not enough on their own to cause the full range of symptoms seen in individuals with PWS. However, they do seem to be involved in the development of overeating and obesity.

There is evidence to suggest that mutations in the MAGEL2 gene may contribute to the characteristic weight gain seen in individuals with PWS. Overeating is linked to a malfunction in the hypothalamic arcuate nucleus, which is where neuropeptide Y (NPY), agouti-related peptide (AgRP), proopiomelanocortin (POMC), and leptin interact to regulate food intake and body weight. NPY and AgRP stimulate food intake, while POMC works to suppress it. In experimental models, a loss of MAGEL2 expression disrupts the normal response of POMC neurons to leptin, leading to increased food intake and uncontrolled fat storage (154, 155). A study by Schaaf et al. identified specific point mutations in the paternal allele of MAGEL2 in four individuals with PWS, where weight gain was a prominent feature along with muscle weakness, developmental delays, and hypogonadism (81).

Kanber and colleagues described a patient with deletions in MKRN3, MAGEL2, and NDN, exhibiting only obesity, developmental delay, and a high pain threshold as the primary clinical criteria for PWS (14). Regarding the series of long non-coding RNAs (lncRNAs) which can be involved in the regulation of gene expression at transcriptional and post-transcriptional levels in PWS, within the long SNURF-SNRPN transcript, there are a series of Small Nucleolar RNAs (snoRNAs) thought to participate in DNA methylation, alternative splicing, and post-transcriptional regulation, such as SNORD116 and SNORD115.

The SNORD116 cluster is crucial in the PWS phenotype. As reported, SNORD116 is involved in controlling NPY neuronal functions, and thus food intake and energy homeostasis. Experimental models on Snord116-KO mice showed PWS features such as hyperphagia (156–159). De Smith et al. (160) reported a 19-year-old male with hyperphagia and severe obesity, mild intellectual disability, and hypogonadism with a 187 kb deletion that included SNORD116.

The SNORD115 gene contains a complementary sequence of 18 nucleotides that matches the mRNA for the serotonin receptor 5-HT2C. Studies on 5-HT2C receptor knockout mice have shown that lack of SNORD115 protein may cause problems with the 5-HT2C receptor, leading to abnormal eating behavior and late-onset obesity (161, 162). Additionally, aside from SNURF-SNRPN and the SNORD gene family, the potential role of NPAP1 in obesity development should be considered (162). Kanber et al. found two patients with deletions in NPAP1, SNURF-SNRPN, and the SNORD genes, who exhibited major clinical signs of PWS, including obesity (14).

In summary, although data linking genotype and obesity in human studies is limited, these findings underscore the importance of taking a comprehensive approach to studying phenotypes throughout life.

## 10 Metabolic syndrome

The metabolic syndrome (MetS) is a common complication of overweight/obesity, leading to T2DM and cardiovascular disease. Diagnosis requires meeting at least 3 of the following criteria: central obesity, arterial hypertension, high triglyceride levels, altered glucose metabolism, and low HDL cholesterol levels (163). Insulin resistance (IR) and obesity are thought to be central factors in the development of MetS (163, 164). Identifying predictive factors for MetS may improve treatment outcomes and prevent severe complications.

Individuals with PWS appear to have a healthier metabolic profile compared to those with essential obesity. This is due to their distribution of subcutaneous fat, higher insulin sensitivity, increased levels of adiponectin and HDL cholesterol, lower rates of fatty liver disease, and reduced cytokines (165–170). However, PWS patients may have higher blood glucose and blood pressure levels, with no significant differences in insulin or insulin resistance compared to controls (171, 172). Additionally, PWS individuals are more likely to have altered glucose metabolism and T2DM (173), although the exact mechanisms are still unknown. The prevalence of MetS in PWS adults and children is 34% and 7% respectively (172, 174), potentially contributing to the reported early mortality rates in the PWS population (171, 175).

Several studies have compared non-syndromic obesity and PWS, but there is limited data available on the clinical features associated with specific PWS genotypes. In particular, information on the genotype-phenotype characterization of MertS and its characteristics is fragmented.

It has been found that adipocyte size is increased in PWS compared to obese controls, with variations observed in different genotypic subclasses (176). Patients with a del15q genotype tend to have higher BMI (137, 176, 177) and HbA1c levels, despite similar values of glycemia, insulinemia, insulin resistance, body composition, metabolic profile, adipocyte size, resting energy expenditure, hyperphagia score, and levels of ghrelin compared to those with mUPD, suggesting a possible association between mUPD genotype and a more adverse metabolic profile, even when adjusted for BMI. The most common form of dyslipidemia in PWS patients, occurring in about 50% of cases, appears to be a decrease in HDL cholesterol, with no significant difference between genetic subtypes (137). Additionally, data from a study involving 108 PWS subjects indicated that the del15q group had a lower risk for low HDL cholesterol and a trend towards a lower risk for MetS compared to non-deleted patients (172). These findings suggest that the del15q genotype group may have the healthiest metabolic profile among adult PWS individuals.

Talebizadeh et al. (168) assessed the body fat composition, IR, leptin, and lipid profile in 55 individuals with PWS and 18 obese controls. They found that in obese individuals, there was a direct correlation between insulin levels and weight, as well as between BMI and subcutaneous fat area. However, these correlations were not observed in the PWS group. On the other hand, PWS patients showed a strong direct correlation between insulin levels and fat area (both visceral and subcutaneous). Specifically, visceral fat and insulin levels were significantly related in the del15q group. Additionally, visceral fat area and triglycerides were directly correlated with the age of individuals in the obese control group but not in the PWS groups. In the del15q and mUPD groups, a positive correlation was found between glucose and triglyceride levels. In the del15q group, there was also a positive correlation between cholesterol and glucose, triglycerides and visceral fat area, insulin and visceral fat area, insulin and triglycerides, and insulin and total cholesterol. These findings suggest a different and specific metabolic profile, at least for one genetic subtype.

Adiponectin is a hormone that sensitizes insulin, and low levels of adiponectin are associated with obesity and MetS. Patients with PWS have higher levels of adiponectin compared to obese individuals, regardless of their physical characteristics (178). This was shown in some recent studies (169, 174, 179) involving different numbers of PWS patients. These studies found no statistical differences in parameters such as insulin, glucose, HOMA, triglycerides, HDL, BMI, and blood pressure among different PWS genotypes.

Irisin regulates glucose levels and insulin sensitivity by promoting glucose uptake and glycogenolysis, as well as reducing gluconeogenesis. It may have a role in obesity and MetS through controlling body weight and regulating the accumulation of white adipose tissue. In a study comparing 25 PWS patients and 25 obese individuals, irisin levels were significantly lower in PWS patients with a del15q compared to obese controls, while patients with a mUPD did not show significant differences compared to controls (180) (**Table 4**).

**Table 4:** Summary of the studies about MetS in PWS.

Author, year	Study design and purpose	Study population	Outcomes	Further considerations
Talebizadeh, 2005 (168)	Cross-sectional study; To examine difference in metabolic profile between PWS vs OC subjects	37 PWS: 20 del15q, age 22.4 ± 7.4 y, 17 mUPD, age 24.2 ± 9.7 y, 18 OC: 25.9 ± 13.3 y,	Positive correlation in PWS del15q group between: - Cholesterol and glucose; - Triglyceride and VFA; - VFA and insulin; - Insulin and triglyceride; - Insulin and total cholesterol. Positive correlation in PWS del15q and mUPD group between glucose and triglyceride;	possible different VFA regulation between PWS and simple obesity subjects. Lower Insulin resistance in PWS.
Kennedy, 2006 (169)	Cross-sectional study; To examine diabetes and cardiovascular risk in PWS subjects compared with OC subjects.	20 PWS: 13 del15q, 7 mUPD; age: 27.7 ± 10.3. 14 OC: age: 26.9 ± 11.4 y	No relevant differences in adiponectin and other variables between PWS del15q and PWS mUPD subjects.	Higher adiponectin levels and less insulin resistance (proportionate to the obesity status) in PWS compared with OC subjects
Brambilla, 2011 (174)	Cross-sectional study; To estimate the frequency of MetS and its components in PWS pediatric subjects	109 PWS (50 obese and 59 non-obese): 58 del15q, 31 mUPD, 20 MET+; age: obese 11 ± 8.1 y; non-obese 10,5 ± 6 y 4 T2DM; 69 GHt. 96 OC: age: 10 ± 5.8 y	No differences in evaluated parameters between PWS genotypes.	Low MetS frequency in non-obese PWS. Similar MetS frequency in obese PWS and OC
Grugni, 2013 (172)	Cross-sectional study; To estimate the occurrence of MetS and its components in PWS adult subjects	108 PWS (87 obese and 21 non-obese): 73 del15q; 27 mUPD; 2 TRAS; 6 MET+ age: obese 26 ± 9 y; non-obese 21 ± 9 y. 23 T2DM 85 OC: age: 28 ± 8 y	Lower risk in PWS del15q for low HDL Lower MetS risk in PWS del15q among genotypes	healthier MetS parameters in non-obese PWS compared with obese PWS and OC.
Lacroix, 2015 (176)	Cross-sectional study; To compare several MetS and obesity parameters between PWS and OC subjects	42 PWS: 27 del15q; 14 mUPD; 1 IMPRINTING MUTATION. (34 MET+ excluded from analysis); age: 25.5 ± 8.9 y; 10 T2DM 42 OC age: 27.5 ± 9.0 y	Lower BMI in PWS mUPD compared with PWS del15q. No differences in the body compositions or metabolic phenotypes or Adipocyte volume between the genotypes (thus, higher relative values in PWS mUPD normalized for BMI). No difference in transcriptomic signature between the genotypes	Lower trunk FM and better metabolic profile in PWS. Significantly higher adipocyte size in PWS vs OCs.
Coupaye, 2016 (137)	Observational study; To compare body composition and metabolic profile between PWS genotype subgroup.	73 PWS: 47 del15q 26.5 ± 9.9 y; 27 mUPD 23.7 ± 6.9 y; Age: 25.5 ± 8.9 y; 14 T2DM	Significantly higher BMI in del15q subjects Significantly higher HbA1c in del15q subjects without diabetes, compared with mUPD patients. No differences in endocrine profile between del15q and mUPD. Higher proportion of percent body fat, adipocyte size and insulin resistance (related to BMI) in UPD compared with del15q. Lower BMI, percentage of body fat and adipocyte size in del15q subjects treated with GH, compared with mUPD	. Decreased HDL as the most frequent type of dyslipidaemia, with no difference between genetic subtypes
Laurier, 2015 (177)	Retrospective study; To analyze medical, psychosocial and social features of PWS subjects according to gender, age, and genotype.	154 PWS: 101 del15q, 24 mUPD, 3 IMPRINTIG DEFECT, 23 MET+, 3 TRAS; age: 28.4.	Significantly higher BMI in del15q group compared with non-del15q group.	
Marzullo, 2020 (179)	Cross-sectional study To evaluate MetS parameters, serum ureic acids and its metabolic effect in PWS subjects compared with OCs.	89 PWS: 67 del15q, 21 mUPD, 1 MET+; age: 28.4 ± 8.7 y 180 OC age: 29.8 ± 7.5 y	No differences in the evaluated parameters between PWS genotypes.	Lower serum uremic acid levels in PWS compared with OC.
Mai, 2022 (180)	Cross sectional study To explore the role of circulating irisin in relation to the metabolic profile and body composition in obese pediatric population with and without PWS	25 PWS: 15 del15q, 10 mUPD. age: 11.1 ± 0.6 y 25 OC age: 12.6 ± 0.7 y	significantly lower irisin levels in del15q group, compared with OC.	Lower irisin levels in PWS compared with OC, Strong association between irisin with insulin resistance, C-peptide and insulin OGTT 120’ levels.

BMI, body mass index; del15q, deletion of chromosome 15; FM, Fat mass; GHt, GH treatment; GIP, glucose-dependent insulinotropic polypeptide; GLP1, glucagon-like peptide-1; HbA1c, glycosylated hemoglobin; HDL, High density lipoprotein; HOMA, homeostasis model assessment; hs-CRP, high sensitivity C-reactive protein; MET+, positive methylation test, cryptogenic; MetS, Metabolic syndrome; mUPD, maternal uniparental dysomy; OC, obese control; OGTT, Oral Glucose Tolerance Test; QUICKI, quantitative insulin sensitivity check index; SFA, Subcutaneous fat area; TRAS, de novo translocation involving chromosome 15; T2DM, type 2 diabetes mellitus; VFA, visceral fat area; y, years.

## 11 Glucose metabolism

Obesity plays an important role in the risk of T2DM in individuals with PWS, as in the general population (171, 173, 181). However, the prevalence of T2DM in PWSs tends to be lower than in obese controls (165, 182). The relationship between adipose tissue (AT) and glucose metabolism in PWS remains largely unknown, although several studies have observed indices of lower IR compared to controls (166, 183, 184). The possible explanations for the higher insulin sensitivity in PWS could be the relatively lower visceral adipose tissue (VAT) and higher subcutaneous adipose tissue (SAT) than BMI-matched controls, as well as the predominant accumulation of subcutaneous fat in the trunk and proximal limbs, with a lower ratio of trunk-to-appendicular fat (167, 185, 186); the downregulation of many IR-associated genes in the AT of subjects with PWS, including those encoding proinflammatory markers (176); the elevated levels of ghrelin (187) and adiponectin (183); the impaired GH secretion (110); the lower high-sensitivity C-reactive protein and IL-6 concentrations (166); the different composition of the gut microbiota compared to patients with obesity (188). Therefore, the pathophysiological mechanisms underlying T2DM in PWS might be different from those involved in common obesity. Since IR is an important risk factor for the development of T2DM, the relatively lower IR could be a protective factor against the risk of developing T2DM in PWS patients, which is common in adults but rarely develops in childhood. More generally, the prevalence of T2DM in adults with PWS ranges from 11 to 25%, compared to 5 to 7% in the general population (173, 189), while it is reported less than 2% in patients younger than 18 years of age (190). On the other hand, data from a cohort of 74 children with PWS showed the presence of impaired glucose tolerance by OGTT in 4% of subjects, but no T2DM (36). In this context, the different genotypes (del15q or mUPD) do not appear to be correlated with the development of altered glucose homeostasis in PWS (174). Furthermore, no genotype-phenotype correlation studies seem to emerge from the literature review. In addition, it should be noted that rare cases of T1DM have also been described (191).

The average age at diagnosis of T2DM in PWSs is about 20 years (171, 173, 191). The onset of T2DM is closely related to the presence of obesity in PWS, the latter related to an insufficient and delayed satiety response, due to uncontrolled food intake mechanisms caused by a hypothalamic dysfunction (21, 192). Currently, the only available control for hyperphagia for most people with PWS is external supervision by mentors with constant supervision, which is challenging and distressing for both caregivers and patients. Access to food must be limited to ensure low caloric intake, considering that children with PWS require 20-30% less energy intake than healthy children of the same age (193). Healthy lifestyles are at the forefront of management of altered glucose metabolism in PWS, with metformin as the first-line drug therapy. When monotherapy is not sufficient, sulfonylureas, meglitinides, thiazolidinediones, dipeptidyl peptidase-4 (DPP-4) inhibitors, glucagon-like peptide-1 (GLP-1) agonists (liraglutide, exenatide, semaglutide, dulaglutide) or insulin can be combined (171). In addition, sodium-glucose cotransporter-2 (SGLT-2) inhibitors have been used in adult patients with PWS in uncontrolled situations (194). Taking into consideration the pediatric age, only insulin and metformin are approved for children (over 10 years of age), but recently GLP-1 receptor agonists (liraglutide) have been introduced for the treatment of T2DM. Moreover, liraglutide has been approved for use in obese non-diabetic children older than 12 years. Some studies have reported the beneficial effects of incretin mimetics as an effective therapy for hyperphagia and obesity in PWS, considering its potential effects on ghrelin suppression, central appetite suppression (through its action on pro-opiomelanocortin POMC/CART neurons and cocaine- and amphetamine-regulated transcripts in the arcuate nucleus (195),), increased energy expenditure (EE) and stimulation of insulin secretion, which could counterbalance the hypoinsulinemia reported in PWS (196–200). However, the data are still conflicting and other authors have reported no effect of liraglutide on weight loss either in children and adolescents with PWS (201).

The recommendations for screening for T2DM and metabolic syndrome are like the general guidelines for subjects with obesity (119). Patients with PWS should be screened for hemoglobin A1C, lipid profile, and transaminases at baseline and then annually. Regarding common diabetes-related complications, there are surprisingly few reports in the literature (202), and their follow-up is identical to that used in the general population.

Although new drugs such as GLP-1 agonists show potential in controlling weight, appetite, and blood glucose in subjects with PWS, diet, physical activity, and behavioral modifications remain the main strategies to promote good metabolic health in these patients.

## 12 Puberty and fertility in females

PWS is characterized by dysfunction of the hypothalamus, leading to multiple endocrine disorders, including hypogonadism (203, 204). In female patients, the clinical manifestation of hypogonadism can vary widely and may change at different stages of life (204, 205). While newborns with PWS often have underdeveloped clitoris and labia minora, puberty and breast development typically start on time. However, the progression of puberty is slow, and incomplete development is commonly seen later in life. Most patients experience primary amenorrhea, but some may have spontaneous menarche at a much older age (around 20 years), followed by secondary amenorrhea or irregular periods (73, 203, 204). Overall, the prevalence of hypogonadism in adult females with PWS ranges from 54 to 100% (73, 203, 204).

Hypogonadism in females with PWS is believed to be primarily caused by dysfunction of the hypothalamus. However, more recent studies suggest that primary ovarian dysfunction also plays a role (206, 207). In some patients, a combination of central and peripheral hypogonadism may be present, making them unique from individuals with more typical disorders (207, 208). Some authors have reported extremely low or undetectable inhibin B levels alongside normal or subnormal Anti Mullerian Hormone (AMH) levels, indicating a specific defect in folliculogenesis. However, a small subgroup of women have detectable inhibin B levels, potentially indicating partial ovarian follicular development and serving as a possible marker of fertility (205–208). Pregnancy has been documented in a small number of women with genetically confirmed PWS (204, 209, 210). Additionally, precocious puberty has rarely been observed in females with PWS (211). MKRN3, a gene located in the critical region of PWS, has been implicated in regulating pubertal development in typical individuals. MKRN3 deficiency has been identified as a common genetic cause of CPP, with different prevalence rates in sporadic (0.5-17-5%) versus familial (9-46%) cases (212, 213). Research on the influence of MKRN3 on pubertal development in females with PWS is limited.

Mariani et al. (214) evaluated MKRN3 protein levels in 80 individuals with PWS at various stages of pubertal development. They found that MKRN3 levels were measurable in 49 patients with PWS, but did not correlate with phenotype, genotype, or gonadotropin levels. This suggests that different genetic mechanisms at the tissue level may be involved in pubertal development in individuals with PWS (213, 214).

Many studies have investigated the potential correlation between genetic background and hypogonadal dysfunction in individuals with PWS, but no data have shown an impact of the various genetic defects (such as 15q11-13 paternal deletion, mUPD, or imprinting defect) on pubertal development and fertility (73, 203, 204). Despite a lack of connections between PWS genotype and puberty in humans, research on animal models replicating PWS has suggested that certain genes may be involved in hypothalamic-ovary dysfunction.

The human chromosome 15q11-q13, also known as mouse chromosome 7C, is an imprinting domain regulated by bipartite imprinting centers (IC). Studies on this chromosome region in the PWS mouse model have revealed varying expression and methylation in different tissues (215). Specifically, research by Mapendano et al. (216) has demonstrated abundant expression of the IC transcript in the brain and ovaries of the mouse model, particularly in granulosa cells of developing oocytes. In regards to the Necdin gene, which has been proposed as a potential candidate for PWS in humans due to its lack of expression in the brain, the mouse knockout model exhibited impaired development of GnRH neurons (217). Taken together, these preliminary findings from experimental models suggest a potential influence of gene expression and methylation at the brain and oocyte/follicle levels on pubertal development in individuals with PWS.

## 13 Puberty and fertility in males

PWS presents many challenges, with hypogonadism being identified as the most common hormonal deficiency among affected individuals. Studies by Partsch et al., Matsuyama et al., and Pellikaan et al. have reported that the prevalence of hypogonadism in adult males with PWS ranges from 57% to 100% (218–220). Driscoll et al. (221) have emphasized the significant impact of hypogonadism in PWS, leading to issues such as genital hypoplasia, incomplete pubertal development, and infertility in most cases.

The development of hypogonadism in males with PWS is diverse, with a variety of causes from central to peripheral hypogonadism. Hirsch et al. and Radicioni et al. have highlighted the occurrence of peripheral hypogonadism, a combination of central and peripheral forms, and pure hypothalamic deficit in some cases (206, 222). Interestingly, there is no established correlation between genotype and phenotype, as reported by Lecka-Ambroziack and Pellikaan et al. (71, 220).

Hypogonadism affects males with PWS of all ages. In infancy, it is common to observe cryptorchidism, scrotal hypoplasia, and a short penile length (175). Eiholzer et al. recommend human chorionic gonadotropin (hCG) treatment for males with PWS and cryptorchidism, as it can lead to lower testes and improved genital size before urologic surgery (223). Interestingly, cryptorchidism does not seem to be linked to gonadotropin levels, and there is no correlation between the age at orchidopexy and inhibin B levels, suggesting cryptorchidism does not play a major role in hypergonadotropic hypogonadism (203).

During puberty, males with PWS commonly experience incomplete and delayed development, alongside frequent precocious adrenarche. Besides hypothalamic dysfunction, primary testicular involvement (especially in the seminiferous epithelium) and moderate Leydig cell dysfunction all contribute to abnormal pubertal development in males with PWS (224, 225). Testicular histology often shows reduced or absent spermatogonia, with a progressive degeneration of germ cells during puberty (226). Lecka-Ambroziack, Linnemann et al., Siemensma et al. discuss the intricacies of pubertal development in PWS, with premature adrenarche seen in around 30% of cases. This could potentially lead to early puberty and compromised adult height without treatment (22, 67, 72, 227). In males with PWS, premature pubarche is associated with early pubic hair growth, elevated levels of serum dehydroepiandrosterone sulfate or serum dehydroepiandrosterone, and advanced bone age. These factors might reduce the positive effects of growth hormone on adult height (67).

While hypogonadism is the most common change in the pituitary-gonadal axis, CPP, though rare, has been documented in children with PWS and could be linked to loss-of-function variations in the MKRN3 gene at the 15q11-q13 locus (228, 229). The MKRN3 gene is widely recognized as a cause of CPP in the general population (230). Normally, *MKRN3* is believed to prevent the start of puberty before adolescence at high levels in the brain. A deletion of the MKRN3 allele from the father in PWS can lead to early puberty. Alternatively, high levels of 17-hydroxyprogesterone and DHEA sulfate were found in patients with CPP, suggesting elevated adrenal androgen levels as previously reported in PWS. Therefore, a combination of a father-derived MKRN3 allele deletion, basal adrenarche, and hypothalamic-pituitary acceleration may play a role in the early onset of precocious puberty (229). The type of hypogonadism - hypogonadotropic, hypergonadotropic, or a mix of these - only becomes apparent in late adolescence or early adulthood and appears to stabilize after the age of 20 years (225).

## 14 Discussion

PWS is a disorder caused by the lack of expression of certain genes on chromosome 15. This can be due to different genetic mechanisms, such as deletion of the paternal chromosome 15q11-q13, maternal uniparental disomy for chromosome 15, abnormalities of the imprinting center, or translocations involving chromosome 15. The main characteristic of PWS is hypothalamic dysfunction, which leads to overeating, body temperature instability, increased pain tolerance, and pituitary issues resulting in various endocrine disorders. Autoimmunity could contribute, at least in part, to the hypothalamus-pituitary axis impairment in these individuals, in addition to the genetically determined dysfunction (47). However, we cannot exclude that anti-pituitary antibodies could be an “epiphenomenon”.

Researchers have looked into whether the symptoms of PWS differ based on the underlying genetic cause. It has been observed that individuals with PWS due to deletion often experience sleep problems and speech difficulties (231) more frequently, with those with type I deletion displaying more compulsive behaviors and performing better academically than those with type II deletion (232, 233). In contrast, individuals with mUPD tend to have more neurodevelopmental impairments, including autistic features and behavioral issues, but typically demonstrate higher IQ levels compared to those with deletion of the critical region of chromosome 15 (16, 153, 232–234). These findings suggest that different genetic backgrounds contribute to the variation in symptoms, especially when it comes to neurodevelopmental aspects.

Prompted by this evidence, we reviewed the literature on endocrine features in PWS to evaluate whether a genotype-phenotype correlation can be established. We considered all the endocrine disorders described in this syndrome and concluded that the available data do not support such a correlation, except for some minor features.

Literature data indicate that PWS patients have lower birth weight and a higher prevalence of preterm delivery and SGA (24) compared to the general population, and a tendency for post-term birth (32). Lower birth weight has been inconsistently described in newborns carrying the 15q11.2-q13 deletion (26–28, 31, 34). Whether perinatal characteristics are specific to genotype remains unclear, and further research is needed to clarify this issue, irrespective of pregnancy management.

Thyroid disorders are quite common in these individuals, and the dysregulation of the hypothalamic-pituitary-thyroid axis is the most frequent cause (35–45). Studies that have attempted to find a correlation between different genotypes and phenotypes have been unsuccessful, suggesting that monitoring thyroid function should be done at any age (**Supplementary Table 1**).

Similar to the thyroid gland, the adrenal gland may also produce insufficient cortisol, with the most common cause being central dysfunction. This can result in primary and secondary ACTH deficiency, especially during stressful situations. Cortisol levels were found to be lower in children with PWS due to del15q compared to those with UPD (52), but this was not consistent with other data from children (54) and adults (55). There was no correlation between genotype and fasting cortisol levels (64, 65), although DHEA-S levels were found to be higher in individuals with del15q, leading to a higher rate of premature adrenarche in the paper by Gaston et al. (70) but not in the paper by Siemensma et al. (67). It is important to note that the diagnostic tests used to assess adrenal function are not consistent across studies, which may explain some discrepancies in findings. Fortunately, individuals with PWS typically do not experience adrenal crisis and do not require glucocorticoid treatment. There is still ongoing debate about the use of hydrocortisone for critical illness and surgery in these individuals, with no consensus reached yet.

Growth mechanisms are considered as one of the most important research fields in PWS, both because of short stature and the availability of a treatment for over 20 years now (100, 107–109). GHD has a 3 times higher incidence in patients with mUPD than in those with del15q. These latter patients also show a correlation between GH response to standard stimulation tests and BMI, as well as a better GH response to standard stimulation compared to mUPD patients (108). Current studies on IGF binding-protein cleavage and IGF bioavailability could offer new insights into the physiopathology of the GH-IGF-1 axis and linear growth mechanisms (235). In individuals with mUPD or ID, GH therapy appears to prevent cognitive decline, suggesting that cognitive skills may be more impaired in these individuals due to more severe GH secretion insufficiency.

Currently, growth hormone therapy is discontinued when adolescents reach adulthood. In a recent letter to the editor, Höybye et al., on behalf of the Clinical and Scientific Advisory Board of The International Prader-Willi Syndrome Organisation (IPSWO), proposed that growth hormone treatment should be extended to adults with genetically confirmed PWS as well. This is an intriguing proposal, as it is widely recognized that in adulthood, growth hormone has positive effects on maintaining normal metabolism and body composition, physical fitness, and beneficial effects on cardiovascular risk factors, and ultimately, quality of life in these individuals (236).

The absence of the sense of satiety is a key characteristic of PWS, resulting in excessive eating and weight gain, leading to MetS and T2DM. Although there is no evidence supporting a correlation between genotype and body fat distribution or obesity, research has suggested that certain genetic mechanisms involving the MKRN3 and MAGEL2 genes, as well as Small Nucleolar RNAs, may play a role in controlling body weight (14, 81, 156–162). While these genes are likely involved in the development of excessive eating and obesity in individuals with PWS, none of them fully explain the entire PWS phenotype.

Obesity is the initial stage leading to MetS and T2DM. Despite being incomplete (**Table 4**), the available data on genotype-phenotype correlation suggests that individuals with PWS who have the del15q genetic subtype often have a higher BMI compared to other genetic subtypes. However, they also tend to have a healthier metabolic profile, which reduces the risk of complications from MetS (133, 176, 177). Additionally, compulsive eating behavior appears to be more common in individuals with mUPD (144), while those with the del15q subtype seem to respond better to multidisciplinary rehabilitation interventions (180) due to a lower prevalence of psychiatric disorders (5, 237, 238).

Various studies have shown no differences in MetS parameters among different genetic subtypes within large population studies (169, 174, 179). This implies that the regulation of body weight and the development of MetS are more likely due to psychiatric, behavioral, and developmental factors rather than solely genetics. Surprisingly, the incidence of T2DM in PWS individuals is lower than expected (165, 182), indicating they have a healthier metabolic profile compared to weight-matched healthy individuals.

Further investigation into the food-seeking behavior leading to severe obesity could reveal distinctions between individuals with mUPD and del15q, aiding researchers in understanding the extent to which metabolic disorders stem from neurodevelopmental issues or genotype-phenotype correlation. This information can help predict complications and guide early interventions for obesity.

Data regarding the onset of puberty and fertility are inconsistent, and a growing amount of data is emphasizing the importance of new genes in controlling puberty. It can be said that there is no clear link between the genes identified in the critical region of PWS and delayed puberty and/or hypogonadism. Additional studies, whether *in vitro* or using animal models, as well as pre-clinical and clinical studies involving humans, would be beneficial in further understanding this form of hypogonadism.

## 15 Conclusions

In conclusion, the available data on endocrine disorders associated with PWS suggest that there is no clear genotype-phenotype association. Only some features of the GH-IGF-1 axis appear to be dependent on the different genotypes. However, a limitation of the analyzed papers is that the cohorts are not homogeneous, which could partially account for the contrasting results from different papers. Another potential limitation in drawing conclusions about genotype-phenotype associations is the small number of patients recruited in many of the referenced studies. Original research based on a very large cohort of people living with PWS, aiming to investigate the genotype-phenotype correlation, would be appreciated as they would shed new light on these features, supporting clinicians in optimizing follow-up.

## Author contributions

SM: Writing – review & editing, Writing – original draft. LZ: Writing – review & editing, Writing – original draft. SV: Writing – review & editing, Writing – original draft. VC: Writing – original draft, Supervision, Data curation. AC: Writing – original draft, Supervision, Data curation. LD: Writing – original draft, Supervision, Data curation. MF: Writing – original draft, Supervision, Data curation. DF: Writing – original draft, Supervision, Data curation. LG: Writing – original draft, Supervision, Data curation. ML: Writing – original draft, Supervision, Data curation. EM: Writing – original draft, Supervision, Data curation. RP: Writing – original draft, Supervision, Data curation. ES: Writing – original draft, Supervision, Data curation. MS: Writing – original draft, Supervision, Data curation. MW: Writing – original draft, Supervision, Data curation. SB: Writing – original draft, Supervision, Data curation. CB: Writing – original draft, Supervision, Data curation. RB: Writing – original draft, Supervision, Data curation. MC: Writing – original draft, Supervision, Data curation. DC: Writing – original draft, Supervision, Data curation. FD: Writing – original draft, Supervision, Data curation. RF: Writing – original draft, Supervision, Data curation. NF: Writing – original draft, Supervision, Data curation. NI: Writing – original draft, Supervision, Data curation. LM: Writing – original draft, Supervision, Data curation. CM: Writing – original draft, Supervision, Data curation. VR: Writing – original draft, Supervision, Data curation. CS: Writing – original draft, Supervision, Data curation. GF: Writing – original draft, Resources, Investigation, Funding acquisition. LI: Writing – original draft, Supervision, Data curation. VS: Writing – original draft, Supervision, Funding acquisition, Data curation. AS: Writing – review & editing, Supervision, Project administration, Conceptualization. MG: Writing – review & editing, Supervision, Project administration, Conceptualization. GG: Writing – review & editing, Supervision, Project administration, Investigation. MD: Supervision, Data curation, Conceptualization, Writing – review & editing, Writing – original draft.
