# Supplementary material for: Endocrine features of Prader-Willi syndrome: a narrative review focusing on genotype-phenotype correlation
Source: Front Endocrinol (Lausanne). 2024 Apr 26;15:1382583. doi: 10.3389/fendo.2024.1382583 (PMC11082343; doi:10.3389/fendo.2024.1382583)
Supplement: Supplementary file 1 [file DataSheet_1.pdf]

**Age of life**	**Prevalent pattern of thyroid function**	**Clinical features**	**Recommended timing of thyroid function evaluation**
Neonatal age	Euthyroidism	--	Neonatal screening and, if negative, evaluation of thyroid function within 3 months of life
Early infancy (infant and toddlers)	Central hypothyroidism (30- 72%)	Prolonged jaundice	Within 3 months of life and then every year
		Lethargy<br>Loss of appetite<br>Weak cry<br>Constipation	
Childhood and adolescence	Euthyroidism	--	Every year<br>Before and 3-4 months after GH treatment, then every 6 months during GH treatment
	Central hypothyroidism (2-60%)	Poor growth	
		Weakness<br>Delay of teething<br>Pubertal delay<br>Cognitive delay<br>Constipation<br>Weight gain	
Adult age	Euthyroidism	--	Every year<br>Every 6 months during GH treatment
	Central hypothyroidism (2-30%)	Cognitive impairment	
		Weakness, fatigue<br>Weight gain<br>Irregular periods, including menorrhagia<br>Decreased tolerance to exercise<br>Dry skin, hair loss	

Constipation		
Cold intolerance		
